# No need to detune transmitters in 32‐channel receiver arrays at 7 T

**DOI:** 10.1002/nbm.4491

**Published:** 2021-02-10

**Authors:** Mark Gosselink, Hans Hoogduin, Martijn Froeling, Dennis W. J. Klomp

**Affiliations:** ^1^ Department of Radiology University Medical Center Utrecht Utrecht the Netherlands

**Keywords:** array coils, parallel transmission, RF transmit coils

## Abstract

Ultrahigh field magnetic resonance imaging facilitates high spatiotemporal resolution that benefits from increasing the number of receiver elements. Because high‐density receiver arrays have a relatively small element size compared with the transmitter, a side effect is that such setups cause low flux coupling between the transmitter and receiver. Moreover, when transmitters are designed in a multitransmit configuration, their relative size is much smaller than the sample, reducing coupling to the sample and thereby potentially also the coupling to the receivers. Transmitters are traditionally detuned during reception. In this study, we investigate, for a 32‐channel receiver head array at 7 T, if transmitter detuning of a quadrature birdcage or of an eight‐channel transmit coil can be omitted without substantially sacrificing signal‐to‐noise ratio (SNR). The transmit elements are operated once with and once without detuning and, in the latter, the received signals are either merged with the array or excluded for image reconstruction. For each of the three measurements, SNR and 1/g‐factor maps are investigated. The tuning of the quadrature and eight‐channel transmit coils during signal reception introduced a 10.1% and 6.5% penalty in SNR, respectively, relative to the SNR received with detuned transmitters. When also incorporating the signal of the transmit coils, the SNR was regained to 98.5% or 101.4% for the quadrature and eight‐channel coil, respectively, relative to the detuned transmitters, while the 1/g‐factor maps improved slightly. For the 32‐channel receive coil used the SNR penalty can become negligible when omitting detuning of the transmit coils. This not only simplifies transmit coil designs, potentially increasing their efficiency, but also enables the transmitters to be used as receivers in parallel to the receiver array, thus increasing parallel imaging performance.

## Graphical abstract

Traditionally, transmitter coils are detuned from receivers during acquisition to exclude their noise contribution. However, with high‐density arrays that have smaller elements, this noise contribution can become negligible and can even be overcompensated by acquiring the signal from the tuned transmit elements as well. We demonstrate the effect of a tuned quadrature birdcage and an eight‐channel transmit coil on a 32‐channel receiver array at 7 T in terms of signal‐to‐noise ratio and 1/g‐factor performance when loaded with a human head.

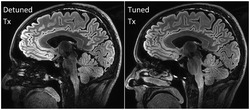

**Abbreviations used**
AP: anterior posterior
CAIPIRINHA: Controlled Aliasing In Parallel Imaging Results IN Higher Acceleration
DC: direct current
FFE: fast field echo
FH: feet head
FLAIR: FLuid‐Attenuated Inversion Recovery
g‐factor: geometry factor
LR: left right
MR: magnetic resonance
MRI: magnetic resonance imaging
MS: multislice
pTx: parallel RF transmission
RF: radio frequency
Rx: receive
SAR: specific absorption rate
SENSE: SENSitivity Encoding
SNR: signal‐to‐noise ratio
TR: repetition time
TRS: transmit receive switch
TSE: turbo spin echo
Tx: transmit

## 1 INTRODUCTION

Ultrahigh field magnetic resonance imaging (MRI) of the human brain provides a detailed window to observe brain pathologies, anatomy, physiology and brain function. Further improvement in the level of details can be obtained by increasing spatiotemporal resolution, which can be obtained with a higher density of receiver elements. For instance, at 3 T, a 96‐channel head array showed superior acceleration performance over traditionally used 32‐channel arrays.^1^ Likewise, a 64‐channel array at 7 T proved to provide substantially improved acceleration performance over a 32‐channel array while preserving intrinsic sensitivity in the center of the brain.^2^ In fact, at higher fields with corresponding higher frequencies, coil sizes can be reduced further while still maintaining a low noise figure.^3^ At element sizes as small as 1 × 2 cm, positioned very close to the human head, tissue loss was shown to be dominant and as such gave a low noise figure.^4^ Simulations using these small elements showed a substantial gain in potential acceleration performance yet would require a 256‐channel receiver setup to cover the entire brain.^5^ Even with the use of improved acceleration techniques such as Controlled Aliasing In Parallel Imaging Results IN Higher Acceleration (CAIPIRINHA), these high‐density receiver arrays show a complementary acceleration performance, as demonstrated at 7 T, albeit in a confined region of the brain, due to the absence of receiver lines in the MRI system.^6^

Despite evidence of increased acceleration performance using high‐density receiver arrays, the number of commercially available receivers is limited. While at 3 T some vendors have provided a platform with up to 128 simultaneous receiver lines,^7^, ^8^ at 7 T this is lagging behind. Indeed, commercially available radio frequency (RF) coils at 7 T go up to only 32 channels, and also at 3 T the full channel count is not used in any commercially available receiver array. Currently, most vendors have provided digital magnetic resonance (MR) signal detection close to the coil port, which makes it relatively easy to upscale the number of receivers and thus potentially not hinder the interfacing of high channel count receiver arrays. Remaining arguments to not have these arrays available may be their complexity in construction and consequently higher cost, especially in combination with multitransmit coils.^9^

Next to enhancing the number of receiver elements to improve precision imaging at 7 T, it is also important to mitigate peak local specific absorption rate (SAR). This allows reduction of the repetition time of RF pulses and also providing more uniform excitation and refocusing. Peak local SAR per unit of time can be reduced by distributing the RF power differently over time causing hotspots to be at different locations over the object during the pulse sequence.^10^ With more transmit elements, the peak SAR can be distributed more effectively in such a way that ultimately global SAR will become the limiting factor.^11^ Moreover, a higher number of transmit elements provides more spatial fidelity in the transmit fields, which can be used to improve uniformity in spin excitation and refocusing, or to facilitate shorter or higher bandwidth RF pulses.^12^ However, multitransmit coils may be considered more complex to design than quadrature birdcages, albeit that very simple dipole transmitters have been shown for body MRI up to 10.5 T.^13^ Such antennas can be placed over local receivers with low mutual inductance and therefore can be designed without detuning circuitry while even contributing to better overall signal‐to‐noise ratio (SNR).^14^

The smaller coil elements in a high channel array have several practical simplifications when compared with larger elements. First, when the individual element is made rigid, their mechanical connection to neighboring elements can be made flexible, making the setup act as a flexible array, without altering the inductance and thus tuning of the elements.^4^ Second, the flux coupled from the transmit coil into the smaller element during receive is less as the surface area is less. Therefore, it reduces the effort in providing perfect active detuning of the transmitter and may even exclude the need for extra safety protection when detuning is malfunctioning. Third, coupling of small coils at a larger absolute distance will be less compared with larger elements, thereby intrinsically improving g‐factor performance.^5^ Fourth, the number of distributed capacitors will be less or even absent, which simplifies the detuning circuitry even further. Fifth, it may even be that due to the reduced flux coupling of the receiver to the transmitter that the transmitter may not be equipped with active detuning while preserving SNR. This last potential simplification is the research topic of this study.

As such we have investigated the necessity to detune transmit coils for the human brain at 7 T without considering geometrical mutual inductive decoupling. The performance of a 32‐channel receiver array is quantified by comparing SNR and 1/g‐factors in detuned versus tuned condition of different transmit coils during reception. Moreover, in a tuned condition, it is investigated if incorporating the signals from the transmit elements with the 32‐channel receiver array can regain the potentially lost SNR. This way, the impact of detuning is shown, which may be extrapolated to further increasing the number of transmit and receive elements, or as demonstrated, used to incorporate the signals from the transmitter elements with the receiver array, but most of all demonstrate simplification in designs of high‐density coil arrays.

## 2 METHODS

### 2.1 Hardware

**FIGURE 1:**
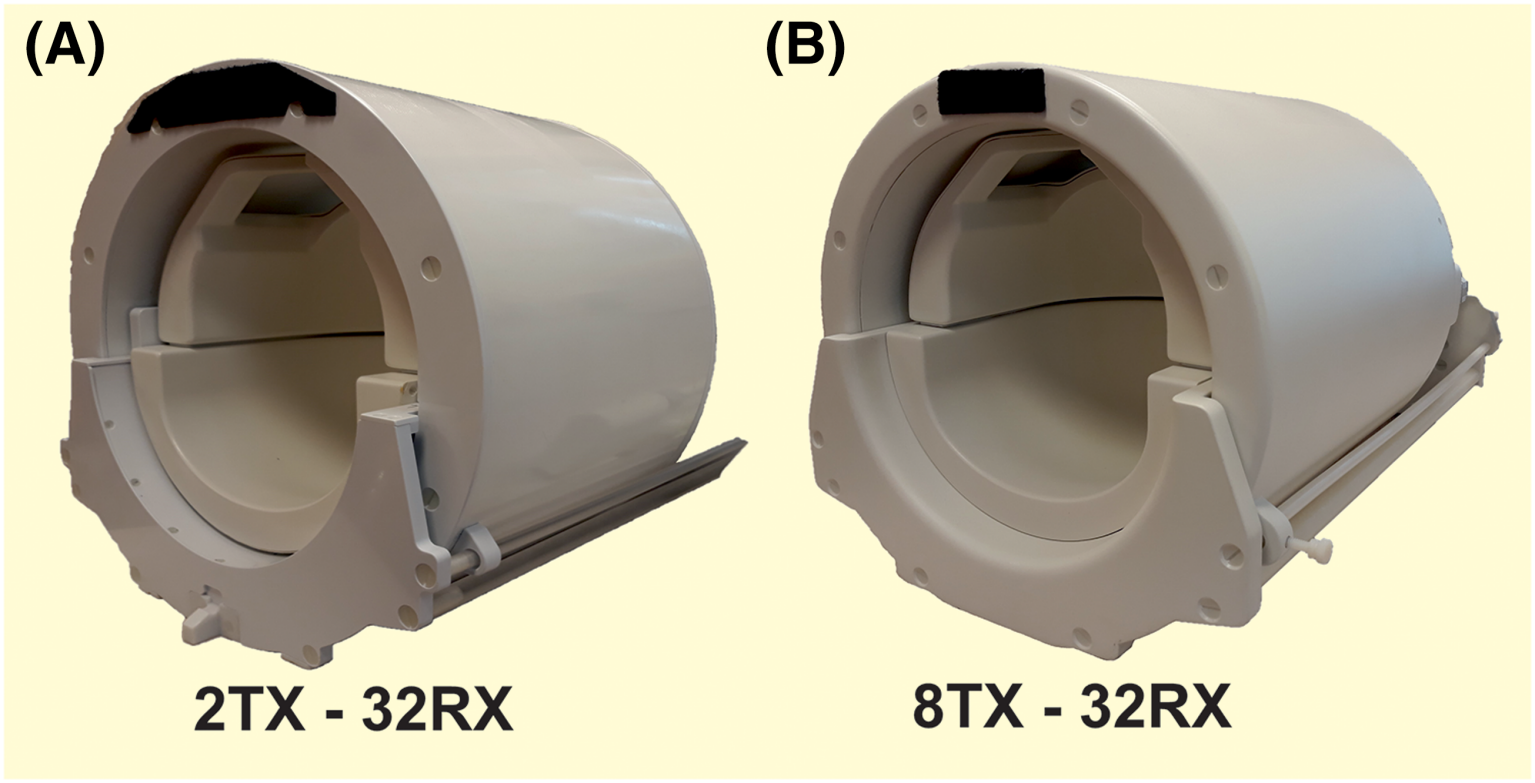
A dual‐channel quadrature volume head‐coil setup combined with a 32‐channel receiver array, A, and an eight‐channel transmit head‐coil with a 32‐channel receive array, B

A whole‐body 7 T MR scanner (Philips Healthcare, Best, the Netherlands) standardly equipped with 32 receive channels and eight parallel RF transmission (pTx) channels was upgraded using digital (dStream) architecture to expand the number of receivers to enable reception with 40 channels simultaneously at 7 T. To evaluate the effect of a tuned transmitter during reception, two widely used RF transmitters were used: a quadrature two‐channel volume head‐coil (Nova Medical, Wilmington, MA, USA) using the classical console and an eight‐channel parallel transmit head‐coil using the upgraded console (Nova Medical). A 32‐channel receiver head‐coil (Nova Medical) was used for receive. Consequently, two setups were investigated: a two‐channel transmitter or transceiver with 32 receivers (2 Tx ‐ 32 Rx or simulated 2 Tx ‐ 34 Rx) and an eight‐channel transmitter or transceiver with a 32‐channel receiver array (8 Tx ‐ 32 Rx or real‐time 8 Tx ‐ 40 Rx) (Figure 1).

The quadrature volume transmitter is a birdcage coil with 16 rods and has a diameter of 29.5 cm and an RF shield (37.0 cm diameter). Active detuning circuits are integrated to detune the coil with a current source (–5 V/200 mA) during acquisition to decouple the coil from the receiver array. The external control to actively drive tuning can be disconnected from the MR system and connected to a permanent voltage source (+12 V) to maintain the tuned condition of the quadrature transmit coil. The parallel eight‐channel transmit coil has a similar inner diameter (28.5 cm) and shield diameter (35.0 cm) to the quadrature volume transmitter; however, the conductor layout has not been made available by the vendor. Each transmit element can be actively tuned with a current source (–5 V/100 mA) during signal reception, yet is not designed to benefit from preamplifier decoupling. For the parallel transmit setup, the tuning control input of the transmit receive switch (TRS) interface is connected to a permanent voltage source (–5 V) to set the driverboard to a tuned state to maintain the tuned condition of the parallel transmit coil.

The 32‐channel receiver head‐coil is an insert that is designed in combination with the aforementioned transmitters. The elements are approximately 8 × 10 cm in size and are organized in two rows of 16 coils each and are mounted on an upper and lower part head‐shaped cup. The coils are overlapped by approximately 10% in the anterior–posterior direction and have a gap of about 2 cm in the transverse direction. The elements are actively detuned during transmit and have short cables that connect to preamplifiers, with incorporating preamplifier decoupling using phase shifters, directly behind the coil. The output of the preamplifiers as well as the power source and detuning lines are interfaced with the MRI system. The detune circuit of each receiver coil is real‐time monitored by the malfunction check of the MRI system.

### 2.2 Measurements

Experiments were performed using a spherical phantom with a diameter of 100 mm (Philips Healthcare). This low conductivity (CuSO_4_ + SH_2_O [60 mg], actetate [2.5 ml], ethanol [5.0 ml], H_3_PO_4_ [4.4 ml]) phantom provides marginal loading of the coil resulting in a low Qunloaded to Qloaded‐ratio of less than 2. Additionally, MRI data were collected from a single male subject (aged 30 years) for the quadrature two‐channel coil setups, and three male subjects (aged 30–46 years) with the eight‐channel transmit coil. The study was approved by the ethical review board of the University Medical Center Utrecht and written informed consent was obtained from all subjects.

Simultaneous data acquisition with all 32 or 40 active channels was obtained with the MRI system for the eight‐channel transmit setup once with the transmitter in detuned state and once in the tuned state. For the quadrature setup, the data had to be acquired subsequently using the 32‐channel receiver array with a detuned transmitter versus a tuned transmitter and repeated once more to redirect the signals of the transmitter to the receive lines of the console to simulate a potential 34‐channel setup. First, B_1_^+^ maps were obtained to ensure identical flip angle distribution between the scans. Then, for SNR comparisons, low flip angle transverse 2D multislice (MS) fast field echo (FFE) scans were obtained (flip angle = 10 degrees; TR = 500 ms; 2 mm isotropic resolution; 256 × 256 × 256 cm^3^ field of view) with two dynamic acquisitions. During the second dynamic scan, the RF and gradients were disabled to acquire a noise‐only scan. Raw complex data per channel were exported for data reconstruction and processing.

Finally, high‐resolution scans were performed using the eight‐channel pTx coil setup in a detuned versus tuned state. For this, a 3D FLuid‐Attenuated Inversion Recovery (FLAIR) turbo spin echo (TSE) sequence was used (flip angle = 90 degrees; TR = 8 s; 0.8 mm isotropic resolution; 240 × 240 × 240 cm^3^ field of view; SENSitivity Encoding [SENSE] = 2 × 2.8).

### 2.3 Postprocessing

Noise correlation matrixes were calculated from the second dynamic noise scan data, which contained more than 2 million samples, with a bandwidth of 128 kHz. The average and standard deviation of the noise correlation matrix were compared between the two setups, as well as under the tuned and detuned conditions of the transmit coil.

First, SNR maps were obtained by using the SNR‐valued reconstruction method of Kellman and McVeigh.^15^ Using a dynamic noise scan for the noise covariance matrix ensures the same scaling for the signal and noise allowing for direct SNR calculation. After calculating the noise covariance matrixes, a noise prewhitening step was applied, which ensures a uniform noise for every channel. Thereafter, sensitivity‐weighted reconstruction using equal noise weighting was performed according to Roemer et al.^16^ A data mask that captures the entire brain was applied to calculate the mean SNR within the subject.

Second, 1/g‐factor maps were calculated by omitting lines from the fully sampled k‐space. The 1/g‐factor maps were obtained using the coil sensitivity maps and the noise covariance matrixes according to Pruessmann et al.^17^ The minimum and average 1/g‐factors were compared between the setups for 2D and 3D parallel imaging accelerations up to 25‐fold (5 × 5) for anterior posterior (AP)‐left right (LR), AP‐feet head (FH) and LR‐FH directions.

## 3 RESULTS

### 3.1 Coil coupling and noise covariance

**FIGURE 2:**
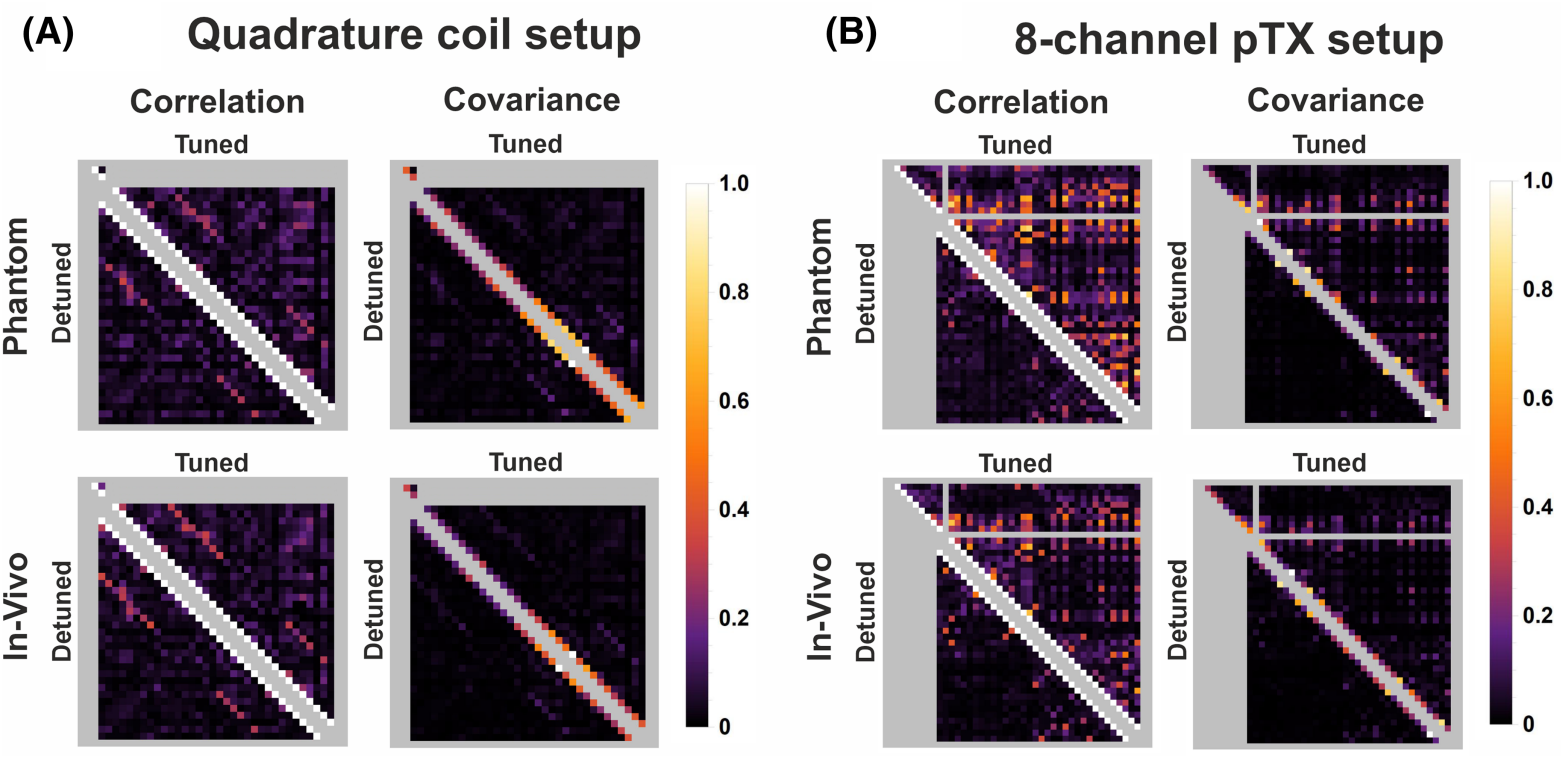
Noise correlation and covariance matrixes for the quadrature coil setup (A) and the eight‐channel parallel radio frequency (RF) transmission (pTx) setup (B). All left lower triangles show the correlation and covariance of the 32‐channel receiver array for a detuned transmitter. All top right‐hand sides show the noise correlation and covariance of transmitter elements, 32‐channel receiver array and, for the eight‐channel pTx setup only, the combination in the tuned state. The quadrature transmitter negligibly affects the covariance of the 32‐channel receiver coil. Larger covariance differences are measured for the pTx transmitter setup in the tuned state compared with a detuned transmitter. The covariance improves when the loading of the coil is increased, as is observed for both setups when the coil is phantom‐loaded versus head‐loaded

The noise correlation and covariance matrixes that include all transmit and receive coil elements are shown in Figure 2. Here, an increased noise correlation in the tuned versus the detuned state of the transmitter can be observed. For the quadrature coil, the noise correlation between the simultaneously active 32 channels shows subtle increases when comparing a detuned with a tuned state of the transmitter. For the marginally loaded eight‐channel transmit coil, the noise correlation is substantially increased. The mean noise correlation values are shown in Table 1. Despite increased correlations, no strong coupling resulting in peak split of the S11 of the transmit elements occurred.

**TABLE 1:** Off‐diagonal noise correlation values

	In vivo mean [range]			Phantom mean [range]		
**2 Tx – 32 Rx**	**All**_**34**_	**Tx**_**2**_	**Rx**_**32**_	**All**_**34**_	**Tx**_**2**_	**Rx**_**32**_
Detuned Tx	‐	‐	0.05 [0.00–0.36]	‐	‐	0.05 [0.00–0.34]
Tuned Tx	‐	0.18 [0.18–0.18]	0.06 [0.00–0.33]	‐	0.04 [0.04–0.04]	0.07 [0.00–0.29]
**8 Tx – 32 Rx**	**All**_**40**_	**Tx**_**8**_	**Rx**_**32**_	**All**_**40**_	**Tx**_**8**_	**Rx**_**32**_
Detuned Tx	‐	‐	0.04 [0.00–0.50]	‐	‐	0.06 [0.00–0.39]
Tuned Tx	0.09 [0.00–0.73]	0.11 [0.02–0.35]	0.09 [0.00–0.72]	0.16 [0.00–0.85]	0.12 [0.01–0.53]	0.16 [0.00–0.85]

Noise correlation values for the quadrature and parallel radio frequency (RF) transmission (pTx) head coil setup for both the tuned and detuned state. Based on the color maps of Figure 2, the mean values of the off‐diagonal entries are separated as all correlations (All), correlation of the transmit elements only (Tx) and correlation of the 32 receiver elements (Rx).

### 3.2 SNR assessment

**FIGURE 3:**
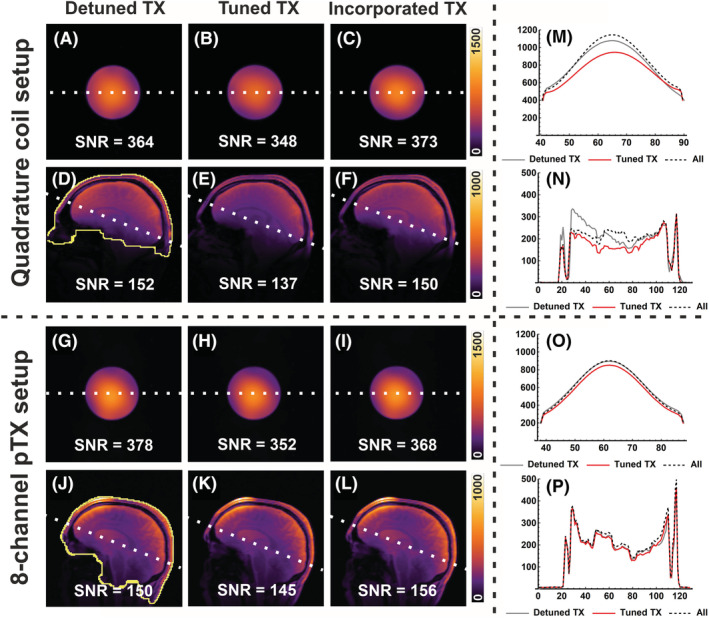
Calculated signal‐to‐noise ratio (SNR) maps of the phantom (A‐C, G‐I) and the human head (D‐F, J‐L) obtained with the 32‐channel receiver array when the transmit coil (quadrature, A‐F, or eight‐channel parallel radio frequency [RF] transmission [pTx], G‐L) is detuned (A, D, G, J) versus tuned state, either without (B, E, H, K) or with the data from the transmitters incorporated (C, F, I, L). The indicated SNR number is calculated as the average SNR over the area indicated by the yellow line. SNR profiles marked with a dashed line for the three states are shown for the phantom SNR maps (quadrature [M], eight‐channel pTx [O]) and the in vivo SNR maps (quadrature [N], 8‐channel pTx [P])

Figure 3 shows the SNR‐valued reconstructions for the phantom and in vivo scans. The baseline SNR for all comparisons is the calculated SNR of the 32‐channel array with a detuned transmitter. For the phantom experiments shown in Figure 3A,B,G,H, the SNR decreased by 4.3% (347.9 to 363.5) and 6.9% (352.0 to 378.2) when the quadrature volume transmitter and eight‐channel transmitter, respectively, were tuned. When the signals from the tuned transmitter coil are incorporated in combination with the local receiver coils, the SNR loss is mostly compensated. In this case, the overall SNR is increased by 2.6% (373.0 to 363.5) for the quadrature setup (Figure 3C) and decreased by 2.8% (367.6 to 378.2) for the eight‐channel transmit setup (Figure 3I).

In Figure 3D,E, a significant SNR reduction is observed towards the center of the brain for the quadrature setup when the transmitter is tuned. The SNR penalty in vivo is larger compared with the phantom measurements, resulting in an SNR loss of 10.1% (136.6 to 152.0). Incorporation of the signals from the transmit coil to the receiver array shows a regain of SNR to 98.5% (149.6 to 152.0) for the quadrature setup (Figure 3F).

The pTx setup shows a subtle overall SNR drop, presumably because of the smaller element size. Quantitative SNR results from the three subjects scanned with the eight‐channel transmitter in tuned state with 32 or 40 channels as receiver are listed in Table 1 and are −7.9% to −3.7% and −0.5% to +3.7%, respectively. The SNR penalty in vivo for the eight‐channel pTx coil is lower compared with the quadrature setup, resulting in an average SNR loss of 6.5% (143.4 to 153.4) when comparing the tuned with the detuned state of the transmitters (Figure 3J,K). Incorporation of the signals from the transmit coil to the receiver array shows a regain of SNR on average to 101.4% (155.5 to 153.4) for the eight‐channel transmitter from the same subject (Figure 3L).

Incorporating the signal from the transmitter contributes mainly to the center. This provides a more homogenous SNR map as, without the signals of the transmitters, the sensitivity of the local receivers decay towards the center. Besides, the larger field of view of the transmitters ensures an increased SNR for the cerebellum and the brain stem.

### 3.3 1/g‐factor evaluation

**FIGURE 4:**
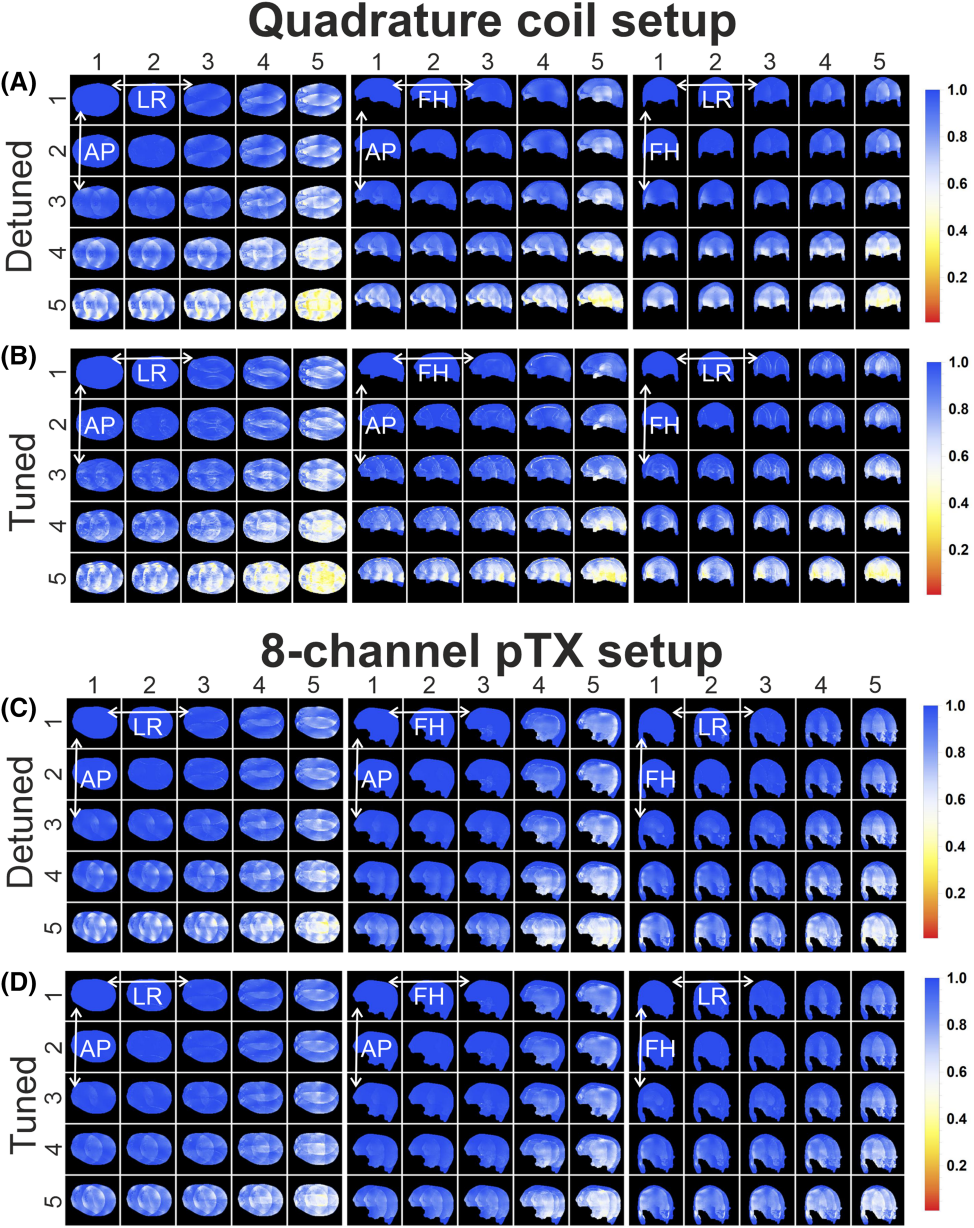
1/g factor maps for accelerations up to 25‐fold (5 × 5) for left right (LR)‐anterior posterior (AP), feet head (FH)‐AP and LR‐FH directions for the quadrature coil setup (A, B) and the eight‐channel parallel radio frequency (RF) transmission (pTx) setup (C, D) in the detuned (A, C) and tuned state (B, D) of the transmitter. Minimal changes are observed whereby the mean 1/g‐factor remains similar for all maps

**FIGURE 5:**
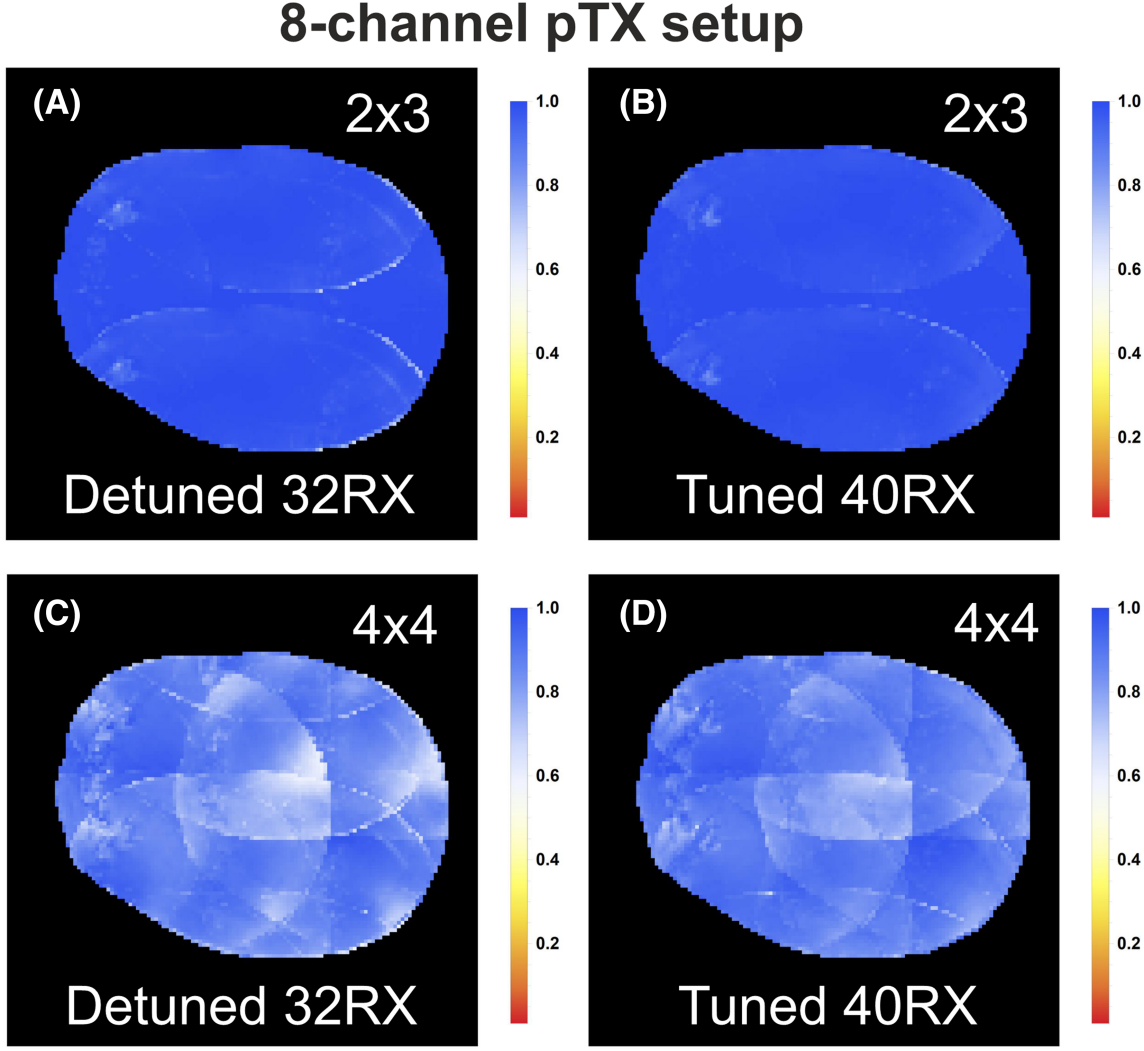
Transverse acceleration maps of the 1/g‐factor results for a 6‐fold (A, B [2 × 3]) and 16‐fold (C, D [4 × 4]) acceleration from one of the three subjects collected with the eight‐channel parallel radio frequency (RF) transmission (pTx) head coil in the detuned (A, C) and tuned state (B, D) of the transmitter. More homogenous 1/g‐factor maps are observed when the transmitter coil is incorporated, resulting in a smaller 1/g‐factor range

The 1/g‐factor maps of the simulated SENSE factors in three orientations are shown in Figure 4. For both setups, the overall SENSE performance is similar for the tuned and detuned coils. However, incorporating the signal of the transmitter elements results in a slightly more homogenous 1/g‐factor map. Figure 5 shows a 16‐fold (4 × 4) transverse acceleration map of the detuned versus tuned state when the signal of the pTx transmitters is incorporated. Although the mean 1/g‐factor is practically unaffected, the range decreases at which the lower bound is improved by 5% (0.77 to 0.81) for all three subjects when incorporating the signal from the transmitters compared with the 1/g‐factor when the transmitter is detuned, as shown in Table 2.

**TABLE 2:** Signal‐to‐noise ratio (SNR) and 1/g‐factor results for the parallel radio frequency (RF) transmission (pTx) head coil

8‐channel pTx coil with 32‐channel receiver array								
SNR	Subject 1		Subject 2		Subject 3		Average	
32‐channel (detuned Tx)	162.3		150.2		147.7		153.4	
32‐channel (tuned Tx)	149.5	−7.9%	144.7	−3.7%	136.0	−7.9%	143.4	−6.5%
40‐channel (tuned Tx)	163.8	+0.9%	155.8	+3.7%	146.9	−0.5%	155.5	+1.4%
**1/g‐factor R = 16 (4 × 4)**								
32‐channel (detuned Tx)	Range [**0.75**–0.94]		Range [**0.77**–0.96]		Range [**0.78**–0.95]			
40‐channel (tuned Tx)	Range [**0.79**–0.95]		Range [**0.81**–0.96]		Range [**0.82**–0.95]		+5.2%	

Abbreviation: AVG, Average.

SNR results of the three subjects collected with the eight‐channel pTx head coil. The results show comparable SNR performances when the signals from the transmit coil are incorporated in the image reconstruction. For one acceleration case (4 × 4), the decreased 1/g‐factor range is shown when comparing the 32‐ with the 40‐channel reconstructed 1/g‐factor maps for the three subjects collected with the eight‐channel pTx head coil. The average 1/g‐factor improvement is based on the minimum 1/g‐factor values in bold.

### 3.4 FLAIR acquisition

**FIGURE 6:**
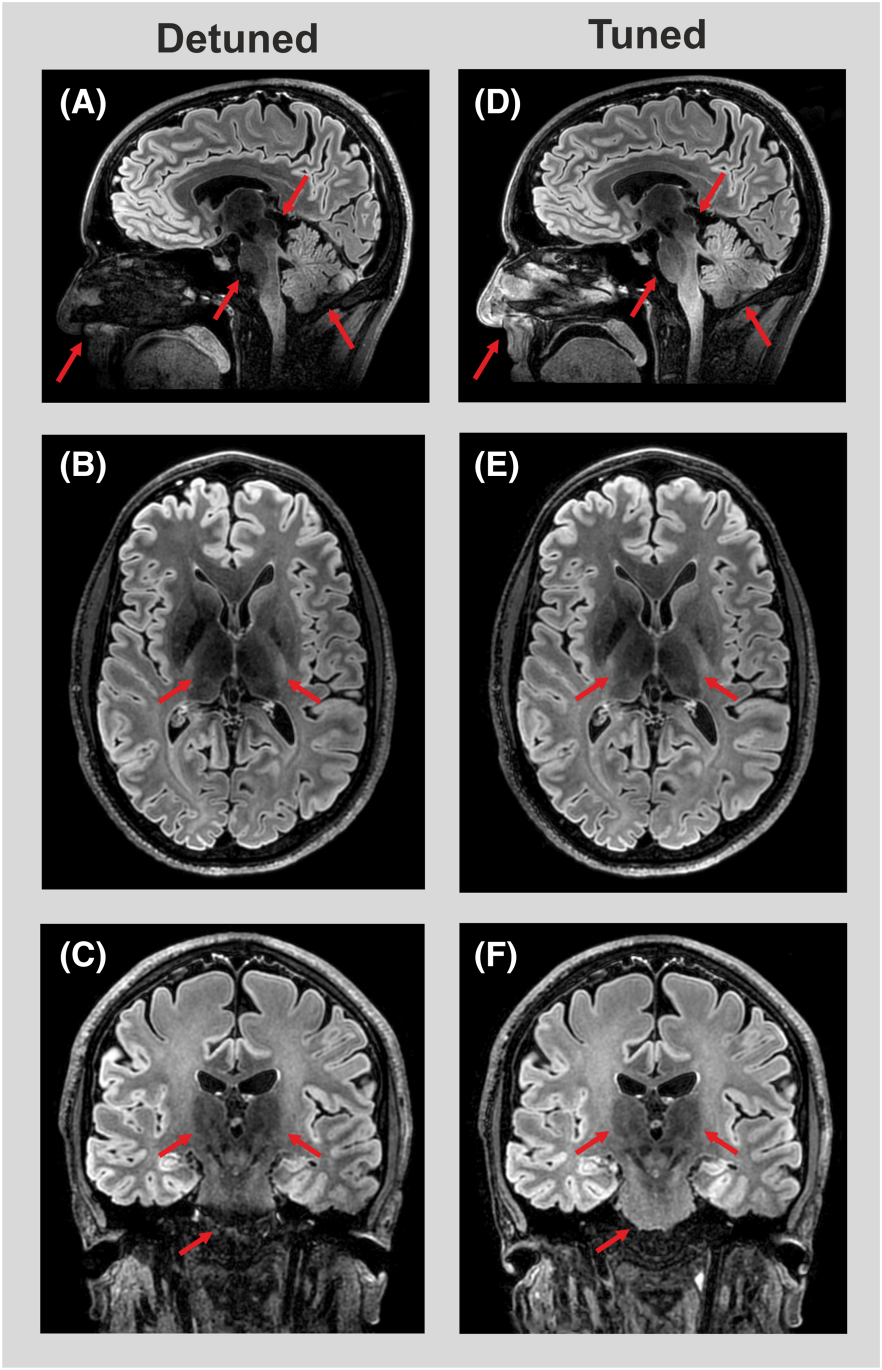
High‐resolution FLuid‐Attenuated Inversion Recovery (FLAIR) images collected with the eight‐channel parallel radio frequency (RF) transmission (pTx) head‐coil using the 32‐channel receiver array (A‐C) in combination with a detuned transmitter compared with all 40 channels, which include the eight tuned transmit channels (D‐F). More homogeneous receive performance is observed when the signal of the eight transmit channels is incorporated, and higher signal‐to‐noise ratio (SNR) is observed outside the brain

The clinically most used FLAIR sequence shows excellent MRI performance (Figure 6). The eight‐channel transmitter, which is configured as a transceiver and merged to the 32‐channel receiver array, shows practically identical to slightly increased image quality compared with the actively detuned eight‐channel transmit coil during reception with the 32‐channel receiver array. Apart from the comparable overall SNR performance within the applied mask, incorporation of the transmitters results in more homogeneous signal distributions, which are visible for this FLAIR image. Besides results showing a more homogeneous image with identical contrast, the features are more visible in the thalamus, pons and the nasal area in the coronal and sagittal views. In addition, no SENSE artifacts were observed in either scan, as was also expected from the 1/g‐factor maps.

## 4 DISCUSSION

This study aimed to investigate the need for detuning the transmitter coil during reception. In most MR systems, transmitter coils are equipped with transmit/receive switches, which enables data acquisition of transmit elements during reception. However, it is generally prohibited to combine this receive capability with reception by the local receive‐only coils due to the risks of coupling too much noise from the transmitters to the local receivers. With subtle software modifications, we could circumvent this restriction and enable the simultaneous reception of signals from the transmit elements as well as from the local receive elements. As such, we measured minimal SNR reduction when the transmitter was not detuned, and practically all SNR could be regained when incorporating the signals from the transmitter elements in the image reconstruction. For this study, the data are processed according to the described SENSE reconstruction method, as the effect may be variable when sum of squares reconstruction is used. Moreover, the slightly increased number of receiver elements used (i.e. 40 rather than 32) that improved minimum 1/g‐factors and the larger homogenous sensitivity profile of the entire transceiver and receiver array resulted in more homogeneously reconstructed FLAIR images.

A combination of transceivers with receivers has been shown to be successful, for instance, by Avdievich et al.,^18^ who merged 18 loop transceivers with 32 receivers for brain MRI at 9.4 T. A combination of dipole and loop elements has been shown by Steensma et al.,^19^ who merged eight dipole transceivers with 16 receivers and Paška et al.,^20^ who merged eight dipole transceivers with an eight‐channel birdcage coil array for body MRI at 7 T. In these designs, care was taken to optimize the entire setup for minimal RF coupling during signal reception with all elements. In our setups, the transmit coil was not designed to be used for simultaneously detecting signals during reception with the local receiver array, yet the coupling between elements remains low for the quadrature setup according to the noise covariance matrixes. The small coupling would cause a minimal impedance change, which may be forgiven considering the relative wide‐band noise‐matching of traditional preamplifiers. Under these conditions, modern reconstruction methods that use noise whitening and sensitivity weighting can cope with coupled coils, hence a positive contribution of the signals from the transmitter elements is observed. Considering the substantially increased RF coupling, as observed in the noise covariation matrix that in contrast to the quadrature setup could only be observed simultaneously for the entire pTx setup, further gain in SNR may be expected when passive decoupling mechanisms are incorporated in the coil design. Although the design details of the commercial coils used in this work are not all known, based on referenced publications of so‐called hybrid coils, translation to other coil applications is feasible. Mainly for new coil designs, optimal decoupling of the transmitter from the receiver can be implemented by design.

A typical transmitter coil design has in‐line PIN diodes with RF chokes, which actively tune the coil with a small direct current (DC) source that forward biases the diode. Depending on the coil design, detune circuits can negatively affect the SNR performance of the coil. The results of this study indicate that these PIN diodes may be excluded in the design, thereby not only simplifying the transmit coil design, but also potentially improving the transmit and receive performance. Besides, because the transmit coil is not optimized for receive, possible new opportunities could be explored to also improve the receive performance. For example, the preamplifier is located on the transmit/receive switch, which in our setup is relatively far away from the coil and coincides with cable losses. Integrated transmit/receive switches could be applied to benefit from preamplifier decoupling and to overcome signal loss between the transmit coil and the preamplifier, resulting in higher SNR.

The relatively large transmitter coil elements have a higher penetration depth compared with the local receiver elements. A combination of the two coil types results in a more uniform receive profile, whereby the contribution of the transmitter coil mostly benefits deep‐lying tissue, as well as in areas where the receiver coil is absent. While our results show similar SNR averaged in the brain when avoiding transmitter detuning, they demonstrate substantial SNR gain in the midbrain and nasal region when including the signals from the transmit elements.

Note that the SNR gain is expected to be dependent on the relative coil element sizes, as observed with the reduced SNR when incorporating the signals from the large birdcage compared with incorporating the signals from the eight‐element transmit coil. Therefore, the consequence of avoiding transmitter detuning is expected to be dependent on the dimensions of the coil elements, both the transmit and receive elements. Isolation between coil elements by ensuring a significant size differential between elements has been shown by Gilbert et al.^21^ Moreover, the effect of coupling to the noise matching of the preamplifiers is tissue load‐dependent, so the effect of avoiding transmitter detuning is expected to be RF frequency‐dependent, implying even more favorable conditions at higher fields. Extrapolating our results to clinical MRI at 3 T that uses a quadrature body coil transmitter, it may be expected that the coupling to even higher channel count receiver arrays could be at an acceptably small level, despite the lower RF frequency compared with 7 T.

Our results, which demonstrate an ability to omit the transmit detuning while maintaining high SNR, are expected to be applicable to a wide range of coil setups at many field strengths. However, when the RF coil coupling is too large, the coil tuning and matching can be affected beyond the optimal noise impedance of the preamps, which will cause a reduction in SNR. Moreover, severe coil coupling will also affect the 1/g‐factor maps, which can compromise the acceleration performance. While coil coupling depends on many variables, in general it can be stated that at lower frequencies, and with relatively larger receiver coils, the RF coil coupling will increase to a point that omitting transmitter detuning will affect the SNR.

## 5 CONCLUSION

Our study has shown that when the signals from the transmit coil are incorporated with the signals from the local receivers, SNR reduction caused by the resonant transmit coil is negligible. Deviating from traditional separation of the transmitter coil to the local receiver coils using transmit detune circuits could be avoided and would simplify the transmit coil design.
